# Preconception Risk Factors for Autism Spectrum Disorder—A Pilot Study

**DOI:** 10.3390/brainsci10050293

**Published:** 2020-05-14

**Authors:** Hankus Magdalena, Kazek Beata, Paprocka Justyna, Kapinos-Gorczyca Agnieszka, Magdalena Szczepara-Fabian, Agata Buczek, Emich-Widera Ewa

**Affiliations:** 1 Department of Pediatric Neurology, Faculty of Medical Science in Katowice, Medical University of Silesia, 42-600 Katowice, Poland; mklhankus@gmail.com (H.M.); marekwidera@wp.pl (E.-W.E.); 2 Persevere—Child Development Support Center, 42-600 Katowice, Poland; beakazek@op.pl; 3 CZP Feniks, Psychiatric Daily Ward for Children and Adolescents, 44-100 Gliwice, Poland; gormasp@o2.pl; 4 Department of Physiotherapy, Center of Early Intervention PSONI, 44-240 Żory, Poland; Magda.lena@op.pl; 5 Department of Neurological Rehabilitation, John Paul II Upper Silesian Child Health Centre, 42-600 Katowice, Poland; neurologopedaagatabuczek@gmail.com

**Keywords:** autism, preconception risk factor

## Abstract

Autism spectrum disorder (ASD) is a neurodevelopmental disorder of multifactorial etiology. Preconception risk factors are still poorly understood. A survey on preconception risk factors for ASD was conducted among parents of 121 ASD patients aged 3–12 years and parents of 100 healthy children aged 3–12 years. The exclusion criteria were as follows: the presence of associated problems such as intellectual disability, epilepsy or other genetic and neurological diseases. Thirteen parameters were considered, a few among which were conception problems, conception with assisted reproductive techniques, the use and duration of oral contraception, the number of previous pregnancies and miscarriages, time since the previous pregnancy (in months), the history of mental illness in the family (including ASD), other chronic diseases in the mother or father and maternal and paternal treatment in specialist outpatient clinics. Three factors statistically significantly increased the risk of developing ASD: mental illness in the mother/mother’s family (35.54% vs. 16.0%, *p* = 0.0002), maternal thyroid disease (16.67% vs. 5.0%, *p* = 0.009) and maternal oral contraception (46.28% vs. 29.0%, *p* = 0.01). Children of mothers with thyroid disorders or with mental illness in relatives should be closely monitored for ASD. Further studies are warranted to assess a potential effect of oral contraception on the development of offspring.

## 1. Introduction

Autism spectrum disorder (ASD) is a neurodevelopmental disorder defined by abnormalities in communication and social interaction, delayed development and repetitive, stereotypical activities [1]. The prevalence of ASD has increased in recent years. It is estimated at 1.34% among 4-year-old children in the USA [2]. The etiology of the disorder is not fully understood. It is assumed that the etiology is most likely multifactorial and the phenotypic expression is influenced by genetic conditions and environmental factors. Some recent studies have demonstrated that the impact of environmental factors can be as high as 40–50% [3,4,5]. These are of great importance because while genetic factors are not currently modifiable, the elimination of potential environmental risk factors could reduce the risk of the manifestation of ASD. The mechanisms of the association between environmental factors and ASD are debated but might include non-causative association (including confounding), gene-related effect, oxidative stress, inflammation, hypoxia/ischemia, endocrine disruption, neurotransmitter alterations, and interference with the signaling pathways [6]. Numerous studies on pregnancy risk factors have been conducted. Additionally, studies have described different parameters influencing fetal neurodevelopment and, consequently, the development of features typical of the ASD phenotype. However, the influence of factors affecting the father and mother before pregnancy is still poorly understood. To date, several studies have shown that maternal overweight and obesity statistically significantly increase the risk of developing ASD in offspring [7,8,9,10,11,12]. In addition, maternal opioid use before pregnancy is an independent risk factor for the development of ASD [13]. In turn, the animal model study showed a positive correlation between preconceptional stressful experiences and the occurrence of an ASD-like phenotype in male offspring [14]. Inconclusive results were obtained in relation to the effect of preconception supplementation with vitamins and folic acid [15,16,17].

The aim of our study was to analyze 13 potential preconception maternal and paternal risk factors for ASD in offspring.

## 2. Material and Methods

### 2.1. Participants

The study group (group 1) consisted of 121 Caucasian children with autism and their biological parents from Silesia (southwestern region of Poland) treated in Katowice or Gliwice (Department of Pediatric Neurology, Child Development Support Center and Psychiatric Daily Ward for Children and Adolescents). The diagnosis was established by a psychiatrist using ADOS-2 (Autism Diagnosis Observation Schedule) as the gold standard observational instrument [18]. The inclusion criteria were as follows: 3–12 years of age and meeting the criteria for ASD. In order to obtain a homogeneous group of patients, which could be defined as the “pure autism group”, strict exclusion criteria have been applied, including the occurrence of related problems such as intellectual disability, epilepsy and other genetic and neurological diseases. 

The reference group (group 2) included 100 Caucasian children with no symptoms of ASD and their biological parents from the same region of Poland. Participants were recruited from primary schools. The inclusion criteria were as follows: 3–12 years of age and the absence of ASD. The exclusion criteria were established as in group 1, i.e., the simultaneous occurrence of an intellectual disability in a child, epilepsy and other genetic and neurological comorbidities.

### 2.2. Methods

The survey was conducted in 2016–2017 among parents of children in both groups. The questionnaire was completed by experienced physicians based on the information obtained from the parent. Participants were informed that their participation in the study was voluntary and that they could withdraw without consequences. The questionnaire used closed questions, while parents were allowed to use the child’s health records. The survey included 13 potential preconception risk factors for ASD in offspring. These factors included conception problems, conception using assisted reproductive techniques, the use and duration of oral contraception, the number of previous pregnancies and miscarriages, time since the previous pregnancy (in months), the history of mental illness in parents and relatives (including ASD) and other chronic diseases in the mother or father (including thyroid disease, cardiovascular disease, ophthalmic disease, and arterial hypertension, epilepsy or diabetes) that occurred before pregnancy, from which the child with ASD was born. Separately, a question was asked about diseases during the pregnancy period (including hypothyroidism). The diseases which rarely occurred in parents were included in the group termed “other”. 

The study was approved by the Ethical Committee of Medical University of Silesia, and approval code No.: KNW/0022/KB1/27/I/15.

### 2.3. Statistical Analyses

The statistical analysis was performed using STATISTICA 10 PL (StatSoft). Comparisons of the distributions of the prevalence of the analyzed risk factors in both groups were performed using Fisher’s exact test. To compare the time interval between the previous pregnancy and the pregnancy from which the child with ASD was born, the U Mann–Whitney was used. The relative risk ratio (RR) and its 95% confidence interval were calculated and its significance was verified for the factors which reached a statistical significance.

## 3. Results

The detailed demographic data on children and their parents are presented in Table 1. Based on the statistical analysis, there was no significant difference in the age and sex between the study and control groups (*p* = 0.20), however, there were some differences in education.

The statistics on the responses to questions on the potential preconception risk factors are included in Table 2, Table 3 and Table 4. Oral contraception was statistically significantly more often used by mothers from group 1 compared with mothers from group 2 (56/121 (46.28%) vs.29/100 (29.0%); *p* = 0.01), while the duration of contraception was insignificant.

A correlation between the occurrence of mental illness in the mother and/or mother’s family and ASD in the child was confirmed (43/121 (35.54%) in group 1 vs. 16/100 (13.0%) in group 2; *p* = 0.0002). Autism spectrum disorder included 8/121 (6.61%) relatives from group 1 and 3/100 (3.0%) relatives from group 2. In turn, mental illness in the father and/or father’s family was found to be insignificant.

In terms of other chronic diseases, maternal thyroid disease had a statistically significant influence on the occurrence of ASD in the offspring (20/120 (16.67%) vs. 5/100 (5.0%); *p* = 0.009). Other diseases in parents were not statistically significant. Similarly, the provision of specialist care to parents did not increase the risk for ASD in the offspring.

Other factors (conception problems, history of previous pregnancy and miscarriage, mean time since the previous pregnancy and conception with assisted reproductive techniques) were observed with a comparable frequency in groups 1 and 2 with no statistically significant influence on the risk of ASD.

## 4. Discussion

The study showed a statistically significant effect of three preconception risk factors for ASD in offspring, i.e., mental illness in the mother/mother’s family, maternal thyroid disease and the use of oral contraception.

There are reports on the correlation between ASD and parental psychiatric disorders. A family history of psychiatric illness was associated with higher odds of ASD in the index persons. An occurrence of ASD, intellectual disability, attention deficit/hyperactivity disorder, obsessive compulsive disorder, schizophrenia and other non-affective psychotic disorders, depression, bipolar disorder and personality disorder was found. The more closely related the affected family member was, the higher the odds were of ASD for the index person. At the same time, ASD without mental retardation was evidently associated with more disorders compare with ASD with an intellectual disability [19]. The association between maternal mental illness and ASD observed in the present study is consistent with this study from the literature.

In an Australian study, compared with mothers with no previous psychiatric contact, those with any psychiatric contact were 2.5-times as likely to have a child with ASD without an intellectual disability and more than twice as likely to have a child with ASD with an associated intellectual disability [20]. Swedish population studies showed a 2-fold higher prevalence of ASD among children of mothers with a psychiatric illness and fathers treated for schizophrenia. Parent diagnoses were based on an inpatient hospital diagnostic evaluation and included schizophrenia, other non-affective psychoses, affective disorders, neurotic and personality disorders and other nonpsychotic disorders, alcohol and drug addiction and abuse, and autism [21]. Similarly, Lauritsen et al. in their study on the Danish population observed that the risk of ASD was twice as high among children whose mothers were diagnosed with a psychiatric disorder compared with children of mothers with no history of psychiatric illness [22]. In addition, the risk of ASD associated with maternal antidepressant exposure during the pre-pregnancy period vs. all unexposed women appeared statistically significantly elevated and was similar in size to that of exposure during pregnancy [23].

The authors did not find data in the literature on the relationship between the occurrence of maternal thyroid disease in the preconception period and the development of ASD in children. However, a statistically significantly increased risk for ASD was observed in the offspring in mothers with hypothyroidism in pregnancy. It was found that the odds of being a probable autistic child at the age of 6-years-old increased almost 4-fold when the mother had severe hypothyroidinemia (defined as 0.03 < TSH < 2.5mIU/L and fT_4_ < 10.99 pmol/L) in early gestation [24]. Maternal hypothyroidism diagnosed and treated for the first time after the birth of the child increased the risk of ASD, whereas no significant association was seen for a maternal diagnosis and treatment prior to the birth of the child [25]. As a risk factor for ASD, autoimmune thyroiditis was also reported in pregnant women. The prevalence of maternal anti-thyroid peroxidase antibodies was significantly increased in pregnancies giving rise to autism cases compared with controls. The odds of autism were increased by nearly 80% among the offspring of mothers who had positive anti-thyroid peroxidase antibodies during pregnancy, compared with mothers negative for this autoantibody. The measures of maternal thyroid hormones did not differ between these groups [26]. Therefore, thyroid disorders in women who plan pregnancy should be effectively treated. More research is warranted to assess the impact of the disorders of thyroid metabolism in the preconception period.

In our study, a history of epilepsy, arterial hypertension and other cardiovascular diseases, diabetes, ophthalmic diseases and other chronic diseases did not have a statistically significant effect on the manifestation of ASD. There are no data in the literature on the impact of these diseases during the preconception period. However, studies have demonstrated an increased risk of ASD in children of mothers with hypertension and/or preeclampsia during pregnancy. The adjusted pooled results of the systematic review and meta-analysis indicated that exposure to maternal gestational hypertension was associated with 35% increased odds of ASD compared with nonexposure [27,28,29]. Similarly, in the case of diabetes, studies have confirmed a statistically significant influence of maternal diabetes on the development of ASD in the offspring, however, without distinguishing preconception glycemic disorders [30,31,32]. Other studies reported that an increased serum glucose level in a pregnant mother was not a risk factor for ASD [29]. Panjwani et al. reported low maternal high-density lipoprotein cholesterol (HDL-C) and above-median maternal plasma branched-chain amino acid concentrations as risk factors [31].

In our study, parental epilepsy was not a risk factor for ASD. However, in the population-based cohort study of Swedish participants, having a first-degree relative with cerebral palsy or epilepsy was associated with a 2-fold increase in the odds for ASD compared with those with unaffected first-degree relatives. The differences in our results may be explained by the fact that the researchers from this publication found a correlation between neurological diseases and ASD with mental retardation, while the group we studied was entirely in the intellectual norm [19].

There are interesting findings related to contraception and its duration as risk factors for ASD. On an animal model, an exposure to progesterone during pregnancy induces ASD-like behavior in the offspring. The researchers used eight kinds of clinically relevant progestins for prenatal exposure in pregnant dams, and the offspring showed autism-like behavior. Therefore, many potential clinical progestin applications (including oral contraceptive pills and preterm birth drugs), may be risk factors for ASD. The mechanism was an estrogen receptor beta (ERβ) suppression in the amygdale [33]. In a previous study, postmortem middle frontal gyrus tissues (13 ASD and 13 control subjects) were examined with the protein levels measurement and gene expression analysis. The gene expression analysis identified a 35% decrease in the ERβ mRNA expression in the middle frontal gyrus of ASD subjects. In addition, a 38% reduction in the aromatase (CYP19A1) mRNA expression was observed in ASD subjects. Significant decreases in ERco-activators were also found. These results provided the evidence of the dysregulation of ERβ and co-factors in the brains of subjects with ASD [34]. Similarly, prenatal levonorgestrel exposure also induced autism-like behavior in the same mechanism, which was demonstrated in the animal model [35]. However, the Chinese population-based case–control epidemiology study revealed that the use of progesterone (to prevent miscarriage and as a contraceptive at the time of conception or prenatal consumption of progestin-contaminated seafood during the first trimester of pregnancy) was strongly associated with the prevalence of ASD. Additionally, in vivo experiments in rats were conducted to further confirm the findings. The subsequent offspring of progesterone-fed dams showed autism-like behavior, which further demonstrated that prenatal progestin exposure may induce ASD [36]. Moreover, a statistically significantly increased risk of ASD was found in children of mothers treated with progesterone due to conception problems. Progesterone exposure during the critical period of fetal life elevated the risk of ASD, possibly reflecting an epigenetic modification [37]. On the contrary, Lyan et al. in their population study observed no correlation between the pre-gravid use of oral contraceptives and the risk of ASD in offspring. Additionally, they presented ambiguous results about the duration (in years) of oral contraceptives: in a retrospective study, the risk of ASD was statistically significantly associated with longer use, though in the prospective sub-group, the oral contraceptives’ duration association was reversed, with a longer duration among mothers of healthy children [38]. Due to the limitations of the available data, it is difficult to draw clear conclusions. Further research is warranted to assess a potential adverse impact of oral contraception on the development of ASD.

The association between the use of assisted reproductive technology and ASD risk in offspring has been explored in several studies, but the results are still inconclusive. The meta-analysis indicated that the use of assisted reproductive technology may be associated with a higher risk of ASD in offspring. An analysis of the total 11 records (3 cohort studies and 8 case–control studies) revealed that the use of ART is associated with a higher percentage of ASD [39]. However, some studies have not demonstrated an increased risk of developing ASD in children conceived using assisted reproductive techniques. In Spain, 231 children conceived by this technique and 208 children conceived naturally under the age of 3 were assessed. No differences were observed in the occurrence of neurodevelopmental disorders (global developmental delay, ASD or speech delay). Based on the analysis of the potential risk factors associated with assisted reproductive techniques, only a correlation between one type of technique (the transfer of frozen embryos) and speech delay was demonstrated, which had not been previously described [40]. Similarly, the study of the Israeli population did not show the effect of in vitro fertilization on the development of ASD compared with the control group of naturally conceived children [37]. Therefore, further prospective, large and high-quality studies are still required.

There are only a few reports on a possible relationship between miscarriage in a previous pregnancy and the manifestation of ASD. In our group of patients with ASD, no correlation was found between a history of miscarriage and the development of ASD in the subsequent children. However, the results of a German study indicated that miscarriage could be a specific risk factor for attention deficit hyperactivity disorder (ADHD) with ASD in children [41].

The authors observed that parents in the reference group are more highly educated than in the study group. So far, it is hard to explain the reason for such an impact. One hypothesis suggests an influence of a healthier lifestyle, however, the authors did not ask about that. Conclusions from various studies assessing the influence of parental education on the risk of autism in offspring are inconclusive [42]. Lee et al. presented findings similar to our results [43]. On the contrary, some studies showed a positive correlation between higher education and ASD [44,45,46]. Therefore, there is a need for further studies.

The strength of this work is a homogeneous study group of children with autism spectrum disorders without additional comorbidities, where a group called “pure autism” was obtained. This is the optimal group that will permit the creation of endophenotypes for further research, and at this stage allows the optimal selection of children for the control group (healthy children in the intellectual norm). Both groups include Caucasian children living in a similar environment, which is extremely important due to the participation in the formation of autism spectrum disorders of genetic and environmental factors. From the researchers’ perspective, the relatively young age of the respondents is important a sit allows the researchers to plan a prospective study in the future, which is a value in itself for population research.

The advantage is also the fact that survey was conducted personally by experienced practitioners and researchers in autistic centers known to children, in which they trust. Parents were not accompanied by children during the study, so they could freely answer the researcher.

A number of limitations should also be noted. The study group for such a common disease is small, where the study at this stage is a form of pilot study, and the conclusions drawn so far will improve the diagnostic tool which is the survey.

The questionnaire is an author’s own questionnaire—it was practically used for the first time. A detailed analysis of the data contained in the questionnaire revealed its disadvantages, including that the questions about psychiatric disorders in parents and in families of autistic children were not sufficiently developed—it would be better to enter closed questions about specific psychiatric diseases entered in the family tree.

Moreover, the data, including sensitive data regarding the family’s health status, were based on information provided by parents: no pregnancy record card or information regarding the health of the patient’s family members were analyzed. Unfortunately, bias due to the interviewers’ subjectivity cannot be ruled out on the responses to some of the questions related to pregnancy and/or family history.

Additionally, most of the children who participated in the study and control groups were unrelated, and while there were also several cases of siblings—due to the small study group—this aspect was not analyzed.

The authors in the presented work use only part of the questionnaire. There are still other questions to be analyzed, including correlations in the clinical picture of ASD or comorbidities in ASD in relation to prenatal factors. Further analysis will perhaps allow to determine endophenotypes and perform more detailed research.

## Figures and Tables

**Table 1:** Demographic data of the study and reference groups.

Factor	Category	Study Group *n* = 121 (100%)	Reference Group *n* = 100 (100%)	Significance Level
children’s sex	male	105 (86.78%)	85 (85.0%)	*p* = 0.70
	female	16 (13.22%)	15 (15.0%)	
children’s age	2–7 years	64 (52.89%)	64 (64.0%)	*p* = 0.10
	8–12 years	57 (47.11)	36 (36.0%)	
mother’s age at the conception		*n* = 120	*n* = 98	
	≤35 years	112 (93.33%)	91 (92.86%)	*p* = 0.55
	>35 years	8 (6.67%)	7 (7.14%)	
mother’s education		*n* = 119	*n* = 90	***p* = 0.02**
	higher	73 (61.34%)	70 (77.78%)	
	secondary *	42 (35.29%)	19 (21.11%)	
	primary *	4 (3.36%)	1 (1.11%)	
father’s age at the conception		*n* = 119	*n* = 97	
	≤35 years	103 (86.55%)	75 (77.32%)	*p* = 0.11
	>35 years	16 (13.45%)	22 (22.68%)	
father’s education		*n* = 118	*n* = 89	***p* = 0.02**
	higher	51 (43.22%)	54 (60.67%)	
	secondary *	50 (42.37%)	33 (37.08%)	
	primary *	17 (14.41%)	2 (2.25%)	

* counted together for the statistical analysis; Statistically significant figures are marked in bold.

**Table 2:** Potential preconception risk factors for autism spectrum disorder (ASD) in mothers of children from the study and reference groups.

Risk Factor	Response	Study Group *n* = 121 (100%)	Reference Group *n* = 100 (100%)	Significance Level
conception problems	Yes	16 (13.22%)	12 (12.0%)	*p* = 0.47
	No	105 (86.78%)	88 (88.0%)	
assisted reproductive techniques	Yes	2 (1.65%)	5 (5.0%)	*p* = 0.25
	No	119 (98.35%)	95 (95.0%)	
another pregnancy	Yes	54 (44.63%)	52 (52.0%)	*p* = 0.28
	No	67 (55.37%)	48 (48.0%)	
time since the previous pregnancy (in months)		*n* = 46	*n* = 47	*p* = 0.53
	mean	55.9	49.1	
	standard deviation	49.9	42.0	
	median	36	29	
	Min–max	3–168	6–144	
previous miscarriages	Yes	12 (9.92%)	11 (11.0%)	*p* = 0.48
	No	109 (90.08%)	89 (89.0%)	
oral contraception	Yes	56 (46.28%)	29 (29.0%)	***p* = 0.01**
	No	65 (53.72%)	71 (71.0%)	
duration of oral contraception		*n* = 56	*n* = 29	*p* = 0.42
	≤1 year	11 (19.64%)	8 (27.59%)	
	>1 year	45 (80.36%)	21 (72.41%)	
mental illness in the mother/mother’s family	absent	78 (64.46%)	84 (84.0%)	***p* = 0.0002**
	ASD *	8 (6.61%)	3 (3.0%)	
	other *	35 (28.93%)	13 (13.0%)	
chronic conditions	thyroid disease	20 (16.67%)	5 (5.0%)	***p* = 0.009**
	cardiovasculardisease	4 (3.33%)	1 (1.0%)	*p* = 0.38
	ophthalmic diseases	3 (2.50%)	2 (2.0%)	*p* = 0.99
	arterial hypertension	4 (3.33%)	0	*p* = 0.13
	epilepsy	2 (1.67%)	0	*p* = 0.50
	diabetes	2 (1.67%)	0	*p* = 0.50
	other	39 (32.50%)	29(29.00%)	*p* = 0.18
care in the specialized outpatient clinic	endocrinology	17 (14.17%)	7 (7.0%)	*p* = 0.13
	cardiology	4 (3.33%)	3 (3.0%)	*p* = 0.99
	ophthalmology	4 (3.33%)	2 (2.0%)	*p* = 0.69
	neurology	9 (7.50%)	3 (3.0%)	*p* = 0.23
	diabetology	0	0	-
	other	18 (15.0%)	13 (13.0%)	*p* = 0.70

* counted together for the statistical analysis; Statistically significant figures are marked in bold.

**Table 3:** Potential preconception risk factors for ASD in fathers of children from the study and reference groups.

Risk Factors	Response	Study Group *n* = 120 (100%)	Reference Group *n* = 100 (100%)	Significance Level
mental illness in the father/father’s family	absent	86 (71.67%)	70 (70.0%)	*p* = 0.88
	ASD *	5 (4.17%)	2 (2.0%)	
	other *	29 (24.17%)	28 (28.0%)	
chronic conditions	thyroid disease	1 (0.83%)	4 (4.0%)	*p* = 0.18
	cardiovascular disease	0	0	-
	ophthalmic diseases	10 (8.33%)	5 (5.0%)	*p* = 0.42
	arterial hypertension	0	3 (3.0%)	*p* = 0.10
	epilepsy	1 (0.83%)	2 (2.0%)	*p* = 0.59
	diabetes	0	0	-
	other	36 (30.00%)	24 (24.0%)	*p* = 0.36
care in the specialized outpatient clinic	endocrinology	1 (0.83%)	4 (4.0%)	*p* = 0.18
	cardiology	2 (1.67%)	3 (3.0%)	*p* = 0.66
	ophthalmology	2 (1.67%)	2 (2.0%)	*p* = 0.99
	neurology	3 (2.50%)	0	*p* = 0.25
	diabetology	0	0	-
	other	14 (11.67%)	13 (13.0%)	*p* = 0.64

* counted together for statistical analysis.

**Table 4:** Relative risks for significant factors.

Factor	Relative Risk (RR)	95% Confidence Interval (CI)	Significance Level
oral contraception	1.38	1.09; 1,74	*p* = 0.007
maternal chronic thyroid disease	1.56	1.23; 1.98	*p* = 0.0003
mental illness in the mother/mother’s family	1.51	1.21; 1.89	*p* = 0.0003
